# Missense variants in *SORT1* are associated with LDL-C in an Amish population

**DOI:** 10.1016/j.jlr.2023.100468

**Published:** 2023-10-31

**Authors:** Kelly A. Mitok, Kathryn L. Schueler, Sarah M. King, Joseph Orr, Kathleen A. Ryan, Mark P. Keller, Ronald M. Krauss, Braxton D. Mitchell, Alan R. Shuldiner, Alan D. Attie

**Affiliations:** 1 Department of Biochemistry, University of Wisconsin-Madison, Madison, WI, USA; 2 Department of Pediatrics, University of California-San Francisco, San Francisco, CA, USA; 3 Department of Medicine, University of Maryland School of Medicine, Baltimore, MD, USA; 4 Regeneron Genetics Center, Tarrytown, NY, USA

**Keywords:** cholesterol/metabolism, dyslipidemias, LDL/metabolism, lipoproteins/metabolism, lipoproteins/receptors, genetic association, genetic linkage, sortilin, *SORT1*, missense variant

## Abstract

Common noncoding variants at the human 1p13.3 locus associated with *SORT1* expression are among those most strongly associated with low-density lipoprotein cholesterol (LDL-C) in human genome-wide association studies. However, validation studies in mice and cell lines have produced variable results regarding the directionality of the effect of *SORT1* on LDL-C. This, together with the fact that the 1p13.3 variants are associated with expression of several genes, has raised the question of whether *SORT1* is the causal gene at this locus. Using whole exome sequencing in members of an Amish population, we identified coding variants in *SORT1* that are associated with increased (rs141749679, K302E) and decreased (rs149456022, Q225H) LDL-C. Further, analysis of plasma lipoprotein particle subclasses by ion mobility in a subset of rs141749679 (K302E) carriers revealed higher levels of large LDL particles compared to noncarriers. In contrast to the effect of these variants in the Amish, the sortilin K302E mutation introduced into a C57BL/6J mouse via CRISPR/Cas9 resulted in decreased non-high-density lipoprotein cholesterol, and the sortilin Q225H mutation did not alter cholesterol levels in mice. This is indicative of different effects of these mutations on cholesterol metabolism in the two species. To our knowledge, this is the first evidence that naturally occurring coding variants in *SORT1* are associated with LDL-C, thus supporting *SORT1* as the gene responsible for the association of the 1p13.3 locus with LDL-C.

The human 1p13.3 locus, which includes the *SORT1* gene, has one of the strongest genetic associations with low-density lipoprotein cholesterol (LDL-C) (*P* = 5 × 10^−324^) (Common Metabolic Diseases Knowledge Portal (CMDKP) - https://hugeamp.org/phenotype.html?phenotype=LDL). Several noncoding variants in linkage disequilibrium (LD) at this locus are associated with an ∼5–8 mg/dl decrease in LDL-C and decreased incidence of coronary artery disease (1). Additionally, this locus is most strongly associated with levels of very small LDL (vsLDL) (2), which are more strongly related to atherosclerosis than large LDL (lgLDL) (3). The variants are associated with hepatic expression of three nearby genes: *CELSR2*, *PSRC1*, and *SORT1* (2, 4, 5). Mediation analysis found that regulation of hepatic *SORT1* expression is primarily responsible for the association of this locus with LDL-C, with *SORT1* levels being negatively correlated with LDL-C (2, 4). These studies suggested that the regulatory mechanism underlying the association of the noncoding variants at the 1p13.3 locus with LDL-C is sortilin-mediated and liver-specific. However, validation studies in mouse and cell models have led to contradictory results, as we summarized in our recent review (1). This raised the possibility that a different gene regulated by the noncoding variants at this locus might be responsible for the association with LDL-C.

The vast majority of genome-wide association study (GWAS) loci are in noncoding regions of the genome. Within these loci are often several highly correlated variants, making it challenging to identify which may be causal (6, 7, 8). In addition to this complexity, there are often multiple genes located at these loci that could plausibly be responsible for the physiological phenotype. A common approach to prioritize candidate genes is to assess the correlations of their expression with variants at a locus. However, a gene with expression most strongly correlated with the variant genotype is not necessarily the responsible gene. The identification of coding variants can provide a direct means for determining causal genes and hypothesizing functional mechanisms. Coding variants often have larger effects on an individual’s traits than noncoding variants, but their rarity makes their contribution to overall heritability small, such that they are often not identified in GWASs (9, 10, 11, 12).

Here, using exome sequencing (ES) in the Old Order Amish founder population of Lancaster County, PA, we identify coding mutations in *SORT1* that are associated with higher (rs141749679, K302E) and lower (rs149456022, Q225H) levels of LDL-C. Carriers of the sortilin K302E mutation have significantly lower levels of lgLDL particles. To study these mutations further, we introduced them into C57BL/6J mice using CRISPR/Cas9 technology. Unexpectedly, the sortilin K302E variant resulted in reduced non-high-density lipoprotein cholesterol (non-HDL-C) and the Q225H variant did not alter cholesterol in mice. We discuss several differences in lipoprotein metabolism between mice and humans that may account for these species-specific effects.

## Materials and methods

### Amish study population

We performed a community-wide survey in 5,987 Old Order Amish individuals aged 18 years and older from Lancaster County, PA, that included a basic physical examination and fasting blood draw during the period 2010–2018 by the University of Maryland School of Medicine’s Amish Research Program (13, 14) (http://www.medschool.umaryland.edu/endocrinology/Amish-Research-Program). The mean age of the individuals used in this study was 41.7 ± 15.4 years, and 44% were male. All study participants provided informed consent. Study protocols were approved by the University of Maryland Institutional Review Board and abide by the Declaration of Helsinki principles.

### Identification of *SORT1* variants

Rs141749679 (sortilin K302E) and rs149456022 (sortilin Q225H) were identified from ES conducted by the Regeneron Genetics Center (Tarrytown, NY) as part of an ongoing collaboration. Exome capture was performed using a slightly modified version of the xGen capture reagent available from Integrated DNA Technologies with some modifications, and the captured libraries were sequenced on the Illumina HiSeq 2500 and NovaSeq 6000 platforms. Captured fragments were sequenced to achieve a minimum of 86% of the target bases covered at 20× or greater coverage variants with call rate <90%. Further technical details of the ES methods have been previously published (15). The rs12740374 (noncoding) variant was genotyped at the Regeneron Genetics Center from the Infinium Global Screening array (Illumina, Inc, San Diego, CA). Following sequencing and genotyping, all samples underwent thorough quality control to remove samples exhibiting high levels of mendelian errors, gender discordance, low coverage (for sequencing), and high genotype missingness.

### Genetic association analyses

Genetic association analyses were performed using linear mixed regression models with genotype as an independent variable and assuming an additive genetic effect. We accounted for relatedness among study subjects by including the genetic relationship matrix in the model as a random effect. Association analyses were carried out using the Mixed Model Analysis for Pedigree and Population software program (http://edn.som.umaryland.edu/mmap/index.php) (16). All association analyses were adjusted for age, sex, sub-study, and measurement protocol (as needed). HDL-C, LDL-C, and triglyceride (TG) were also adjusted for rs5742904 (APOB R3527Q) and rs76353203 (APOC3 R19∗), two variants that are enriched in the Amish population and are highly associated with lipid levels. APOB R3527Q increases LDL-C ∼75 mg/dl (13) and APOC3 R19∗ is associated with decreased TG and increased HDL-C (17) in the Amish. Effect sizes are reported as 1 unit change in trait per allele. To account for the fact that we were testing three variants, we considered genotype differences with *p*-values < 0.017 (0.05/3) to be statistically significant.

### Serum lipid measurements and ion mobility in Amish subjects

**Fig. 1:**
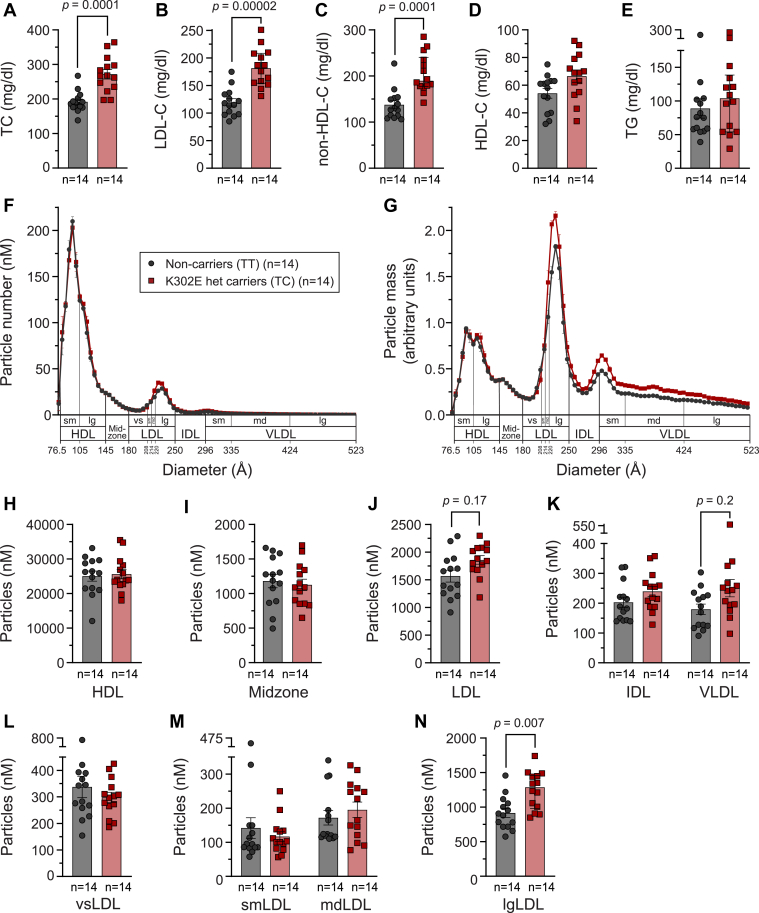
Heterozygote carriers of rs141749679 (sortilin K302E) in the Amish have significantly increased levels of large LDL particles. A: Total cholesterol (TC), (B) HDL cholesterol (HDL-C), (C) non-HDL-cholesterol (non-HDL-C), (D) LDL cholesterol (LDL-C), (E) triglycerides (TG), (F) lipoprotein particle concentration and (G) mass, as analyzed by ion mobility, and concentration of particles in (H) HDL, (I) midzone, (J) LDL, and (K) IDL and VLDL classes and (L) very small LDL (vsLDL), (M) small LDL (smLDL) and medium LDL (mdLDL) and (N) large LDL (lgLDL) subclasses (as defined in supplemental Table S3) in 14 rs141749679 (K302E) heterozygote carriers and 14 age- and sex-matched noncarriers. Serum was collected after an overnight fast. Data in panels A–E, H–K, and L–N were analyzed by three separate repeated measures two-way ANOVAs with Geisser-Greenhouse correction followed by Bonferroni’s multiple comparisons test with individual variances computed for each comparison. All data were log transformed prior to analysis. Data are represented as mean ± SEM.

Serum was harvested from overnight fasting blood and sent to Quest Diagnostics (Horsham, Pennsylvania) for measurement of TC, HDL-C, TG, and glucose. Non-HDL-C was calculated by subtracting HDL-C from TC. LDL-C was estimated using the Friedewald method (18). Prior to ion mobility (IM) measurement of plasma lipoprotein concentration, plasma was treated with 17% ethanol to remove >97% of fibrinogen, and then all lipoproteins were precipitated with 2 mg/ml dextran sulfate and 0.15 M calcium. Precipitated lipoproteins were harvested on paramagnetic particles, washed to remove free salt and proteins (e.g., IgG, albumin, and transferrin), and then resuspended in 25 mM ammonium acetate, as previously described (19). Following isolation, lipoproteins were fractionated and quantified in a single scan using gas-phase electrophoresis (IM), as previously described (20, 21). In the IM “scans” shown in Fig. 1F, G, lipoproteins were grouped into bins, each spanning ∼5 Å diameter. Lipoproteins were then further pooled by summing the total number of particles within specific size ranges that approximately group them into separately defined subclasses that have minimal methodologic and biologic overlap, and as previously characterized (22), to generate the data shown in Fig. 1H–N. supplemental Table S1 shows the lipoprotein subclasses, their size ranges, and nomenclature.

### Generation of sortilin K300E and sortilin Q223H mice

Sortilin K300E and sortilin Q223H mice were made via CRISPR-Cas9 genome-editing by the Gene Editing and Animal Models group at the University of Wisconsin-Madison. For the sortilin K300E mice, the highly specific target sequence GAAACGGCCCCCAAGACCAA was used to introduce rs141749679 (GRCh37.p13 chr 1 NC_000001.10:g.109888432T>C) into a C57BL/6J mouse obtained from the Jackson Laboratory to mutate lysine (K) 300 to glutamic acid (E) in the sortilin protein (Uniprot Q6PHU5-1). For the sortilin Q223H mice, the highly specific target sequence ACAGGTAATCAGAATTCTGA was used to introduce rs149456022 (GRCh37.p13 chr 1 NC_000001.10:g.109897022C>A) into a C57BL/6J mouse obtained from the Jackson Laboratory to mutate glutamine (Q) 223 to histidine (H) in the sortilin protein (Uniprot Q6PHU5-1). All predicted off-targets varied by at least three nucleotides, and no single predicted off-target had an activity prediction score (CFD) higher than 0.5. An in vitro transcription template was generated by overlap-extension PCR with one oligo carrying a 5′ T7 adapter, the target sequence, and a portion of the common gRNA sequence and the other oligo carrying the antisense common gRNA sequence. Following column-purification, the in vitro transcript was transcribed with the MEGAshortscript kit (ThermoFisher), and the resultant gRNA was cleaned with the MEGAclear kit (ThermoFisher), purified with ammonium acetate, washed with 70% ethanol, and resuspended in injection buffer (10 mM Tris-HCl, 0.1 mM EDTA, pH 7.4). A mixture of gRNA (50 ng/μl), ssODN (50 ng/μl), and Cas9 protein (40 ng/μl) were microinjected into the pronuclei of fertilized C57BL/6J one-cell embryos and then implanted into pseudopregnant female B6D2F1 recipients. Resultant pups were genotyped at weaning by PCR of tail DNA. For the sortilin K300E mice, the targeted region was amplified with forward primer TGCAGATTCTCTGTGTATGAT and reverse primer TGCCCAACACATATATCACA. The PCR product was digested with BstXI. The base pair change to create K300E also created a BstXI site. The digest was run on a 2% agarose gel. Wild-type (WT) mice yielded a 471 bp fragment, sortilin K300E homozygous mice yielded 234 bp and 237 bp fragments, and sortilin K300E heterozygous mice yielded 471 bp, 234 bp, and 237 bp fragments. The 471 bp PCR products were also gel-purified and sequenced to identify a founder mouse that contained rs141749679 (g.109888432T>C) and had no other mutations in this 471 bp sequence. rs141749679 was approximately in the center of the 471 bp sequence. For the sortilin Q223H mice, the targeted region was amplified with forward primer GAAGCCGAGGCGGAAGAGTG and reverse primer TTCCAGCAGCAGACATCCGTTC. The PCR product was digested with EcoR1. The base pair change to create Q223H led to the loss of an EcoR1 site. The digest was run on a 2% agarose gel. WT mice yielded 104 bp and 259 bp fragments, sortilin Q223H homozygous mice yielded a 363 bp fragment, and sortilin Q223H heterozygous mice yielded 104 bp, 259 bp, and 363 bp fragments. The 363 bp PCR products were also gel-purified and sequenced to identify a founder mouse that contained rs149456022 (g.109897022C>A) and had no other mutations in this 363 bp sequence. rs149456022 was approximately in the center of the 363 bp sequence. One founder mouse homozygous for either variant was identified and bred to a WT C57BL/6J mouse to generate heterozygotes. Male mice homozygous for either sortilin K300E or sortilin Q223H were compared to age-matched male WT mice produced from the breeding of the respective CRISPR-edited line.

### Animal care and housing

Mice were bred, housed, and cared for in the AAALAC-accredited University of Wisconsin-Madison Biochemistry Department vivarium, and all mouse experiments were conducted in accordance with University of Wisconsin-Madison IACUC-approved protocols. Mice were group housed under temperature- and humidity-controlled conditions, a 12-h light/dark cycle (6 a.m. to 6 p.m.) and received ad libitum access to water and food. Starting at weaning (∼3 weeks of age), mice were fed a Western-style diet high in fat and sucrose (TD.08811, Envigo Teklad Custom Diet) containing 44.6% kcal from fat, 14.7% kcal from protein, 40.7% kcal from carbohydrate, 34% sucrose by weight, and high saturated fatty acids (>60% of total fatty acids).

### Plasma measurements in mice

Mice were fasted for 4-h and bled retro-orbitally to collect plasma whole blood using EDTA as an anticoagulant. Triglyceride was measured using the Triglycerides Reagent from ThermoFisher (TR22421), and cholesterol was measured using the Total Cholesterol Reagent from ThermoFisher (TR13421). To determine cholesterol that is nonprecipitable by heparin-MnCl_2_, plasma was mixed with a solution containing heparin and MnCl_2_ with a final concentration of 220 U/ml heparin and 92 mM Mn^2+^, incubated at 4°C for 20 min, centrifuged at 4°C for 20 min at 1,500 *g*, and the cholesterol concentration of the supernatant was measured using the Total Cholesterol Reagent from ThermoFisher (TR13421). Cholesterol that was precipitable by heparin-MnCl_2_ was calculated by subtracting the nonprecipitable cholesterol from the total cholesterol (TC).

### Statistical methods

Amish genetic association analyses were performed as described above. All other data were analyzed by repeated measures two-way ANOVA with Geisser-Greenhouse correction and Bonferroni’s multiple comparisons test with individual variances computed for each comparison. Log-normally distributed data were log-transformed prior to analysis by ANOVA. ANOVAs were performed using GraphPad Prism 9. Data are represented as mean ± SEM.

## Results

### rs141749679 and rs149456022, resulting in K302E and Q225H in sortilin, are associated with LDL-C in an Amish population

We performed ES on ∼6,000 Old Order Amish residing in Lancaster County, PA, and discovered two rare missense variants in *SORT1* that are associated with LDL-C. The first, rs141749679 (g.109888432T>C, c.A904G, p.K302E), has a minor allele frequency (MAF) of 0.003 in the Amish and in the general European population (Genome Aggregation Database (gnomAD) - https://gnomad.broadinstitute.org/variant/1-109888432-T-C?dataset=gnomad_r2_1). We identified 35 heterozygote (TC) carriers of the K302E variant and no homozygotes. This variant was associated with a substantial 28 mg/dl (13%) increase [95% CI: 12–44 mg/dl (6–21%) increase] in TC (*P* = 0.0006), a 22 mg/dl (17%) increase [95% CI: 8–36 mg/dl (6–27%) increase] in LDL-C (*P* = 0.002), and a 25 mg/dl (17%) increase [95% CI: 10–40 mg/dl (7–27%) increase] in non-HDL-C (*P* = 0.001) and was marginally associated with increased TG (*P* = 0.03) (Table 1). The second missense variant, rs149456022 (g.109897022C>A, c.G675T, p.Q225H), is enriched in the Amish (MAF = 0.026) compared to the general European population (MAF = 0.0002) (gnomAD - https://gnomad.broadinstitute.org/variant/1-109897022-C-A?dataset=gnomad_r2_1). We identified 304 heterozygote (CA) carriers and two homozygotes. This variant was associated with a modest 8 mg/dl (4%) decrease [95% CI: 3–13 mg/dl (1–6%) decrease] in TC (*P* = 0.002), an 8 mg/dl (6%) decrease [95% CI: 4–12 mg/dl (3–9%) decrease] in LDL-C (*P* = 0.0004), and an 8 mg/dl (5%) decrease [95% CI: 3–13 mg/dl (2–9%) decrease] in non-HDL-C (*P* = 0.001) (Table 1). Neither of the two coding variants were associated with HDL-C.

**Table 1:** Association of *SORT1* variants rs141749679 (K302E), rs149456022 (Q225H), and rs12740374 with lipid traits in an Amish population

Variant	Trait	Noncarriers	Het Carriers	Hom Carriers	Effect (95% CI)	*P*
		Mean ± SD or Median (25%, 75%) (mg/dI)				
*rs141749679 (K302E)*		TT (N = 5,904)	TC (N = 34)	CC (N = 0)		
	**TC**	**208 ± 50**	**253 ± 57**		**28 (12–44)**	**0.0006**
	**LDL-C**	**132 ± 46**	**168 ± 50**		**22 (8–36)**	**0.002**
	**non-HDL-C**	**147 ± 49**	**186 ± 50**		**25 (10–40)**	**0.001**
	HDL-C	61 ± 17	68 ± 19		3 (−3 to 9)	0.3
	TG	58 (43, 84)	73 (54, 109)			
	lnTG	4.1 ± 0.5	4.4 ± 0.5		0.2 (0.0–0.4)	0.03

*rs149456022 (Q225H)*		CC (N = 5,633)	CA (N = 303)	AA (N = 2)		
	**TC**	**209 ± 50**	**199 ± 44**	**144 ± 18**	**−8 (**−**3** to −**13)**	**0.002**
	**LDL-C**	**133 ± 46**	**121 ± 42**	**72 ± 28**	**−8 (**−**4** to −**12)**	**0.0004**
	**non-HDL-C**	**148 ± 49**	**135 ± 45**	**85 ± 38**	**−8 (**−**3** to −**13)**	**0.001**
	HDL-C	61 ± 17	64 ± 17	59 ± 20	0.3 (−1.5 to 2.2)	0.7
	TG	58 (43, 85)	54 (42, 79)	62 (30, 94)		
	lnTG	4.1 ± 0.5	4.1 ± 0.5	4.0 ± 0.8	−0.02 (−0.08 to 0.03)	0.4

*rs12740374 (noncoding)*		GG (N = 2,619)	GT (N = 2,649)	TT (N = 685)		
	**TC**	**213 ± 53**	**207 ± 48**	**198 ± 43**	**−5 (**−**3** to −**7)**	**1 × 10**^−**10**^
	**LDL-C**	**137 ± 49**	**131 ± 44**	**122 ± 40**	**−5 (**−**4** to −**6)**	**1 × 10**^−**10**^
	**non-HDL-C**	**148 ± 49**	**135 ± 45**	**85 ± 38**	**−5 (**−**3** to −**7)**	**1 × 10**^−**9**^
	HDL-C	61 ± 17	62 ± 17	62 ± 7	−0.06 (−0.7 to 0.6)	0.9
	TG	58 (44, 84)	58 (43, 85)	57 (42, 82)		
	lnTG	4.1 ± 0.5	4.1 ± 0.5	4.1 ± 0.5	−0.01 (−0.03 to 0.01)	0.3

Abbreviations: Het, heterozygote; Hom, homozygote; LDL-C, low-density lipoprotein cholesterol; non-HDL-C, non-high-density lipoprotein cholesterol; TC, total cholesterol; TG, triglyceride.

Associations were tested using linear mixed regression models. Effect sizes and *P* values are per-allele and adjusted for age, sex, and two coding variants, one in APOB and one in APOC3, previously shown to greatly affect lipid levels in this population (see Methods for details). Effect sizes are in units of mg/dl and include the 95% confidence interval (CI). Genotype-specific data are represented as mean ± SD for normally distributed traits (TC, LDL-C, non-HDL-C, HDL-C, and lnTG) and median (25%, 75%) for nonnormally distributed traits (TG) and are not adjusted for covariates. All traits are in units of mg/dl. TG was natural log (ln) transformed prior to analysis. *P*-values <0.017 (0.05/3, to account for testing three variants) are statistically significant, indicated in bold.

We repeated the association analyses of rs141749679 and rs149456022 with lipids but removed subjects with the rs149456022 variant minor allele (A) from the analysis of rs141749679 and removed subjects with the rs141749679 variant minor allele (C) from the analysis of rs149456022. The results were highly consistent with those shown in Table 1 (i.e., 24 mg/dl higher LDL-C among those with the rs141749679 C allele (*P* = 0.002) and 7 mg/dl lower LDL-C among those with the rs149456022 A allele (*P* = 0.002)).

We compared the effect size on LDL-C of these *SORT1* coding variants with that of the noncoding variant rs12740374 (g.109817590G>T), the putative causal variant for the association of the human 1p13.3 locus with LDL-C in GWAS (CMDKP - https://hugeamp.org/variant.html?variant=1%3A109817590%3AG%3AT) (2, 4). rs12740374 is a common variant in the general European population (MAF = 0.2) (gnomAD - https://gnomad.broadinstitute.org/variant/1-109817590-G-T?dataset=gnomad_r2_1) but is even more prevalent in the Amish (MAF = 0.3), in whom it was associated with a 5 mg/dl (2%) decrease [95% CI: 3–7 mg/dl (1–6%) decrease] in TC (*P* = 1 × 10^−10^), a 5 mg/dl (4%) decrease [95% CI: 4–6 mg/dl (3–9%) decrease] in LDL-C (*P* = 1 × 10^−10^), and a 5 mg/dl (3%) decrease [95% CI: 3–7 mg/dl (2–9%) decrease] in non-HDL-C (*P* = 1 × 10^−9^) (Table 1). This is in line with the ∼5–8 mg/dl decrease previously reported for rs12740374 and other variants in LD at the 1p13.3 locus (4, 23, 24, 25, 26, 27, 28, 29). We performed LD analysis for rs141749679 (K302E), rs149456022 (Q225H), and rs12740374 and found that rs149456022 (Q225H) is in LD with the common noncoding variant rs12740374, while rs141749679 (K302E) is not (supplemental Table S1). When a multivariate model was used to test for the association of each of the three variants with LDL-C, where all three variants were analyzed together, the effect size and strength of the association of rs141749679 (K302E) with LDL-C remained essentially unchanged, while those of rs149456022 (Q225H) decreased (supplemental Table S2), reflecting the results of the LD analysis.

We looked for any association between the rs141749679 (K302E) or rs149456022 (Q225H) variants and lipids in the most recent lipid GWAS, from the Global Lipids Genetics Consortium (30). Phenome-wide association analysis of the aggregated GWAS results across all ancestries revealed that the rs141749679 (K302E) variant is associated with increased LDL-C (beta = 0.04, *P* = 0.002) and that the rs149456022 (Q225H) variant is associated with decreased LDL-C (beta = −0.10, *P* = 0.02) (supplemental Table S3), replicating the association and directionality of the association we observe in the Amish population for these variants. When the data are stratified by ancestry, the K302E variant is significantly associated with LDL-C in European ancestry individuals. K302E carriers of Hispanic or African ancestry have increased LDL-C, and Q223H carriers of European or Hispanic ancestry have decreased LDL-C, but these effects did not reach statistical significance (supplemental Table S3).

### Carriers of the sortilin K302E variant have increased levels of lgLDL particles

To investigate if the K302E variant affects specific subclasses of LDL, we performed IM, a method that directly measures the diameter and concentration of lipoprotein particles (20, 21), in a subset of 14 K302E heterozygote carriers and 14 age- and sex-matched noncarriers. Importantly, the K302E heterozygote carriers in this subset of subjects showed increased TC, LDL-C, and non-HDL-C, and no difference in HDL-C or TG (Fig. 1A–E), in concordance with the association analyses in the full set of subjects. Figure 1F, G show the lipoprotein particle concentration and particle mass, respectively, plotted against particle diameter. Lipoproteins were then pooled by summing the total number of particles within diameter ranges that group the lipoproteins into major classes: HDL, midzone (particles with diameters between those of HDL and LDL), LDL, IDL, and VLDL (Fig. 1H–K), as defined in supplemental Table S4 and as previously described (22). Heterozygote carriers of the K302E variant had a trend for increased LDL and VLDL particles (*P* = 0.17 and 0.2, respectively) (Fig. 1J, K). Division of LDL particles into subclasses that have minimal methodologic and biologic overlap (vsLDL, small LDL, medium LDL, and lgLDL) (Fig. 1L–N) revealed a significant increase in the number of lgLDL particles in K302E carriers compared to noncarriers (*P* = 0.007) (Fig. 1N), which correlated with LDL-C in the 28 subjects (supplemental Fig. S1).

### K302 and Q225 residues are highly conserved and located in the ligand-binding domain of sortilin

**Fig. 2:**
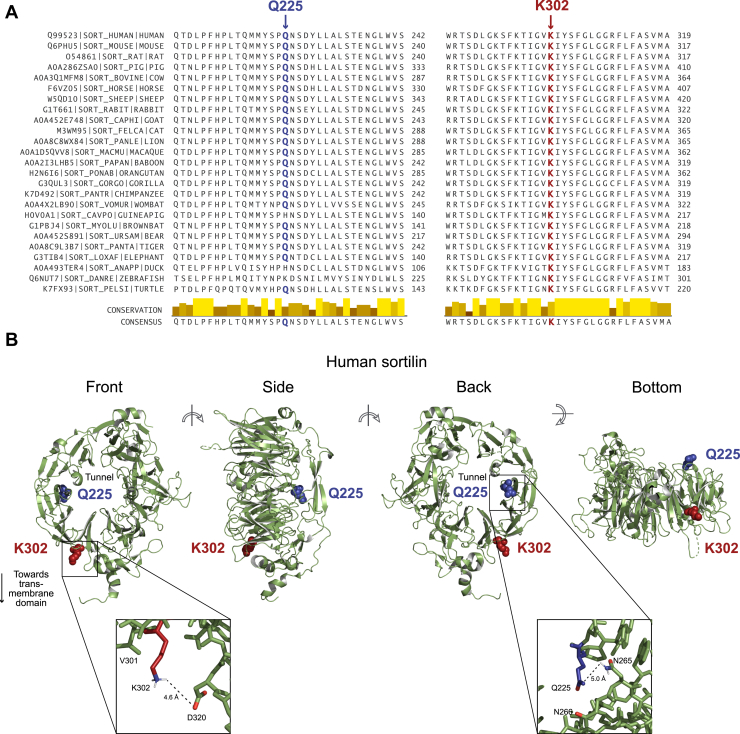
K302 and Q225 are highly conserved residues and are located in the β-propeller domain of sortilin. A: Multiple species alignment of the regions of sortilin containing lysine 302 (K302) and glutamine 225 (Q225) using Clustal Omega (31, 32) and visualized using Jalview (33). B: Structure of the luminal domain of human sortilin as determined by Quistgaard *et al.* (34) (Protein Data Bank 3F6K) and visualized using PyMol (35). K302 and Q225 are highlighted in red and blue, respectively.

K302 and Q225 are highly conserved residues that lie within the 10-bladed β-propeller ligand-binding domain of sortilin (Fig. 2A). K302 lies along the outside edge of the β-propeller, near the transmembrane domain, and is oriented such that it would point toward membrane when sortilin is membrane-anchored inside the cell (Fig. 2B). The side chain nearest to that of K302 is that of D320 and is located 4.6 Å away (Fig. 2B). Q225 is located along the inside edge of the β-propeller, near the narrower “back” opening of the tunnel that passes through the middle of the β-propeller (Fig. 2B). The side chain nearest to that of Q225 is that of N265 and is located 5.0 Å away (Fig. 2B).

### Mice harboring K300E or Q223H do not recapitulate lipid effects seen in the Amish despite a high structural homology of sortilin between the two species

**Fig. 3:**
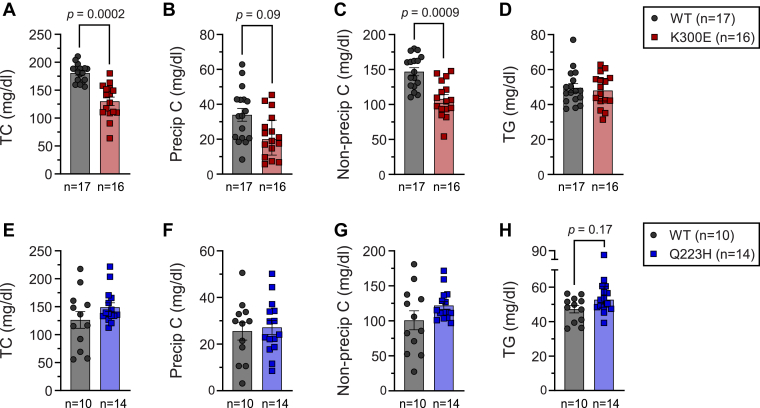
Mice expressing the sortilin K300E variant have decreased TC and heparin-MnCl_2_-precipitable and non-precipitable cholesterol. (A) Total cholesterol (TC), (B) heparin-MnCl_2_-precipitable cholesterol (Precip C), (C) non-heparin-MnCl_2_-precipitable cholesterol (Non-precip C), and (D) triglycerides (TG) in sortilin K300E mice (n = 17 for WT and n = 16 for K300E) and (E) TC, (F) Precip C, (G) Non-precip C, (H) TG in sortilin Q223H mice (n = 10 for WT and n = 14 for Q223H). All mice were male, on a C57BL/6J background, 18 weeks of age, and fed a high-fat, high-sucrose (HF/HS) diet for 15 weeks. Plasma was collected after a 4-h fast. Data in panels A–D and E and F were analyzed by two separate repeated measures two-way ANOVAs with Geisser-Greenhouse correction followed by Bonferroni’s multiple comparisons test with individual variances computed for each comparison. All data were log transformed prior to analysis. Data are represented as mean ± SEM.

Using CRISPR/Cas9, we generated C57BL/6J mouse models expressing either the sortilin K302E variant (K300E in mice) or the Q225H variant (Q223H in mice). WT and CRISPR-edited mice harboring each mutation were fed a high-fat, high-sucrose diet (45% kcal from fat, 34% sucrose by weight) for 15 weeks. We performed heparin-MnCl_2_ precipitation on fasting plasma to determine precipitable and nonprecipitable cholesterol, estimates of non-HDL-C and HDL-C in mice, respectively, a method like that used in the Amish. Surprisingly, sortilin K300E mice had drastically *decreased* TC (*P* = 0.0002) and *decreased* precipitable cholesterol (*P* = 0.09) (Fig. 3A, B), as well as decreased nonprecipitable cholesterol (*P* = 0.0009) (Fig. 3C). This is opposite to our prediction based on the genetic association in the Amish. There was no difference in plasma TG between WT and K300E mice (Fig. 3D). Sortilin Q223H mice had TC, precipitable cholesterol, nonprecipitable cholesterol, and TG levels comparable to that of WT controls (Fig. 3E–H).

**Fig. 4:**
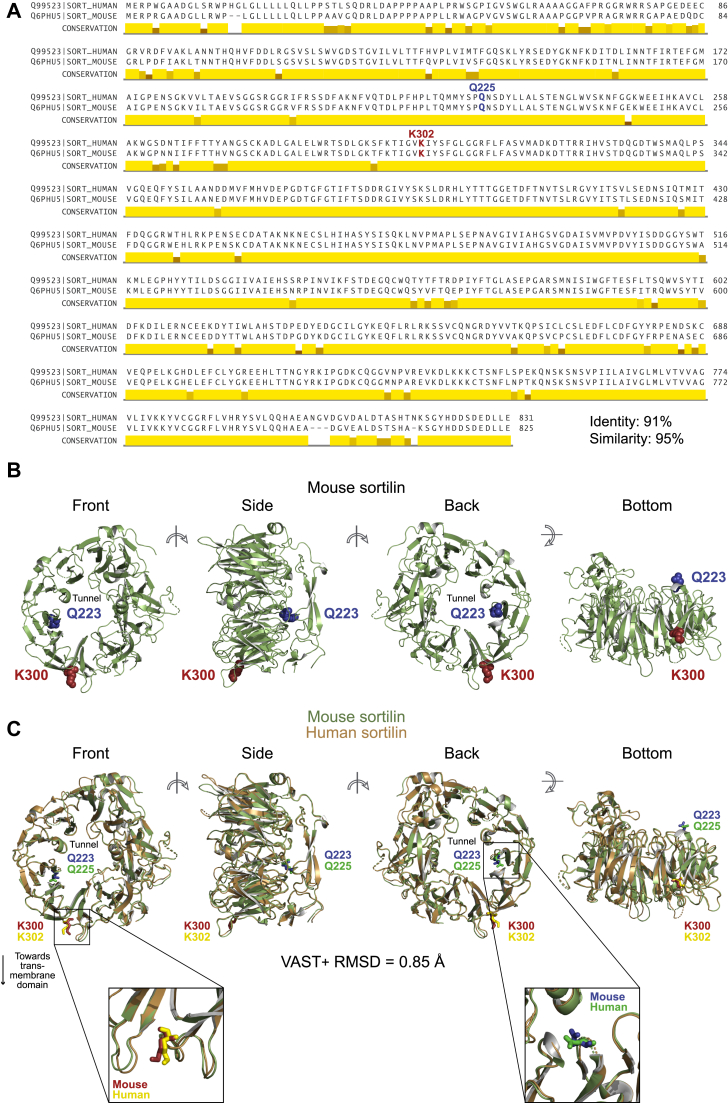
Mouse and human sortilin have high structural homology. A: Alignment of the amino acid sequences of full-length mouse and human sortilin using EMBOSS Needle (36) and visualized using Jalview (33). B: Structure of the luminal domain of mouse sortilin as determined by Leloup *et al.* (37) (Protein Data Bank 5NMR) and visualized using PyMol (35). K300 and Q223 are highlighted in red and blue, respectively. C: Alignment of the structures of the luminal domains of mouse and human sortilin (Protein Data Bank ID 5NMR and 3F6K, respectively) using the Vector Alignment Search Tool VAST+ (38) and visualized using PyMol (35).

The opposing effects of the mutations on cholesterol in mice versus humans is despite a high level of structural homology between mouse and human sortilin. The linear sequence of the full-length mouse and human sortilin proteins are highly conserved, with 91% identity and 95% similarity (Fig. 4A). Further, the structure of the soluble domain of mouse sortilin is strikingly similar to that of human sortilin (Fig. 4B). Alignment of the two structures using the Vector Alignment Search Tool results in a very low root mean square deviation of 0.85 Å, indicating high structural similarity (Fig. 4C). Each of the 588 amino acids modeled in both the mouse and human structures were determined by Vector Alignment Search Tool to be aligned in 3D space.

## Discussion

By conducting ES in the Old Order Amish population of Lancaster County, PA, we identified two coding mutations in *SORT1* associated with LDL-C. To our knowledge, this is the first report that naturally occurring coding mutations in *SORT1* are associated with a cardiovascular disease-related trait. The rs141749679 variant (sortilin K302E) was recently reported to be significantly associated with Alzheimer’s disease (39).

The effect size of the common noncoding regulatory variant rs12740374 on LDL-C is modest compared to that of the rare *SORT1* coding variant rs141749679 (K302E). For several years, the prevailing hypothesis behind the genetic risk of common diseases, such as cardiovascular disease, has been that disease risk alleles with high frequencies cumulatively cause common diseases—the common variant, common disease hypothesis (40). However, more recent work has demonstrated that low-frequency genetic variants with large phenotypic effects can also contribute significantly to complex diseases—the rare variant, common disease hypothesis (12).

In individual GWASs, noncoding variants at the 1p13.3 locus are primarily associated with decreased LDL-C and are not associated with TG (1), similar to what we see in the present study with one of these variants (rs12740374) (Table 1). However, meta-analysis of GWAS datasets reveals a significant association of this locus with decreased TG (*P* = 1 × 10^−14^) (CMDKP - https://hugeamp.org/region.html?chr=1&end=109990540&phenotype=LDL&start=109802190), an effect with the same directionality as that of LDL-C. Previous studies have suggested that the manipulation of *SORT1* expression can affect LDL levels in both VLDL (TG)-dependent and -independent manners (1). Even though the K302E variant was only marginally associated with TG in this study, its effect on LDL-C and TG are also in the same (positive) direction. The opposite directionality of the effect on LDL-C and TG of the K302E variant compared to that of the rs12740374 variant, which is associated with increased hepatic *SORT1* expression, suggests that K302E is a reduced-function mutation. Further studies are required to determine whether the effect of the K302E variant on LDL-C is through an effect on VLDL.

The use of IM allowed us to directly quantify the concentration of lipoprotein particles in different subclasses in Amish individuals. We found that heterozygote carriers of rs141749679 (K302E) had an increased concentration of lgLDL particles and that the effect of the variant was specific to this subclass of LDL. Musunuru *et al.* previously used IM to measure subclasses of lipoprotein particles in carriers of the noncoding variant rs646776 at the 1p13.3 locus, which is in LD with the putative causal variant rs12740374. They reported that homozygote carriers of the rs646776 minor allele had decreased concentrations of all LDL subclasses compared to noncarriers, with the greatest decrease being in vsLDL (20% decrease, *P* = 1.1 × 10^−11^) with progressively smaller decreases for larger LDL subclasses (2). The 1p13.3 noncoding variants are associated with a wider range of LDL particle sizes than the K302E variant, suggesting that the effect of the K302E mutation may be more complex than just a simple reduced function. If sortilin has different binding affinities for different sizes of lipoprotein particles, as has been demonstrated for the LDL receptor (LDLR) (41, 42), a change in these binding affinities induced by the K302E mutation may explain the differential effect of the K302E mutation and the noncoding variant on different sizes of LDL particles.

A major finding of our study is that the sortilin K302E and Q223H mutations have differing effects on cholesterol in mice versus humans. The mouse and human sortilin proteins are highly homologous, both in linear amino acid sequence and structurally. It is therefore highly unlikely that the differences we observe upon introducing these *SORT1* variants into mice are due to the mutations having different effects on the sortilin protein in the two species. Rather, there are important differences in lipoprotein metabolism between humans and mice that may provide an explanation for this. One difference is that mice lack cholesterol ester transfer protein, which in humans mediates transfer of cholesterol from HDL to apoB-containing lipoproteins (43, 44). A second is that the apolipoprotein B mRNA editing enzyme (*APOBEC1*), which is responsible for posttranscriptional production of apolipoprotein B-48 (apoB48) from the apolipoprotein B-100 (*APOB100*) transcript, is expressed in liver and intestine in mice but is only expressed in intestine in humans (45). Thus, unlike humans, mice produce a mixture of liver-derived apoB48- and apoB100-containing particles. While both the LDLR and sortilin bind to apoB100 (46, 47), neither binds to apoB48 (46, 48). Finally, in contrast to humans, mice carry significant amounts of apolipoprotein E (apoE) on LDL particles (49), with much of the hepatic clearance of apoB48-containing LDL occurring through interaction of apoE with LDLR-related protein 1 (50). Sortilin has been shown to bind apoE in the brain (51), making it plausible that it also acts as an apoE receptor in the liver. Given these differences, the fate of LDL in mice is likely to be affected by more proteins than in humans. This, combined with the possibility that K300E or Q223H affects the binding of apoE to sortilin, may contribute to the differential effects of these mutations in mice versus humans.

Both K302 and Q225 are highly conserved residues in sortilin and are located in its ligand-binding domain. Studies that have analyzed the structure of sortilin and/or carried out competitive binding experiments with several of its known ligands have indicated the presence of at least two distinct binding sites within the tunnel of its β-propeller (34, 37, 52, 53). There is evidence for allosteric regulation between the binding sites and for some ligands to span both binding sites (52, 53). Therefore, mutations within in the β-propeller, such as K302E and Q225H, may affect the binding of some ligands to sortilin but not others, allowing for partial loss- or gain-of-function.

The binding of ligands within the tunnel of sortilin’s β-propeller is regulated through dimerization of sortilin at low pH (37). Dimerization occurs along the front of the β-propeller and causes a conformational change that results in collapse of the tunnel (37). Determination of sortilin’s dimerized form predicts a 2-fold axis with the dimer oriented perpendicular to cellular membranes. Based on this proposed orientation, 10 lysine residues reside at the dimer/membrane interface, possibly functioning to stabilize the dimer form by interacting with negatively charged glycolipids in the membrane. K302 is one of the 10 lysine residues at this interface (37). Replacing a positively charged lysine (K) residue with a negatively charged glutamic acid (E) residue may disrupt dimer stability and alter ligand binding, further suggesting a reduced-function effect of this mutation.

A limitation of our study is that it does not address the tissue site of action of sortilin. Our analysis favors the liver as the site of action affecting LDL. Studies following up on the original discovery that the human 1p13.3 locus is associated with LDL-C have found a significant association of the locus with hepatic *SORT1* expression levels, but not with *SORT1* expression in other tissues and cells involved in lipoprotein metabolism, namely white adipose tissue (2), blood vessels (54, 55), monocytes (56), and whole blood (57). However, we remain puzzled by the fact that *SORT1* is expressed at a very low level in the liver and at a far higher level in adipose tissue. Adipose tissue is a major site for VLDL lipolysis by lipoprotein lipase, and one of the earliest ligands identified for sortilin was lipoprotein lipase (58). Thus, it is possible that the functions of sortilin and/or sortilin K302E in adipose tissue may contribute to their effects on lipoprotein metabolism.

In conclusion, this study shows a direct causal association between mutations in the sortilin protein and LDL levels. It provides further granularity to the early studies of Musunuru *et al.* and others, suggesting that the common noncoding locus at human 1p13.3 exerts its effect on LDL through its regulation of *SORT1* expression (2, 46, 59). The identification of coding variants in *SORT1* that are associated with LDL-C in humans presented here provides renewed confidence that *SORT1* is the gene responsible for the strong association between the 1p13.3 locus and LDL-C in human GWAS.

## Data availability

All data described in the manuscript are contained within the manuscript.

## Supplemental data

This article contains supplemental data (20, 21, 22, 30).

## Conflict of interest

The authors declare that they have no conflicts of interest with the contents of this article.
