# Supplementary material for: Missense variants in SORT1 are associated with LDL-C in an Amish population
Source: J Lipid Res. 2023 Oct 31;64(12):100468. doi: 10.1016/j.jlr.2023.100468 (PMC10711479; doi:10.1016/j.jlr.2023.100468)
Supplement: Supplemental Data [file mmc1.pdf]

SUPPLEMENTAL DATA

# Missense variants in *SORT1* are associated with LDL-C in an Amish population

Kelly A. Mitok[1], Kathryn L. Schueler[1], Sarah M. King[2], Joseph Orr[2], Kathleen A. Ryan[3], Mark P. Keller[1], Ronald M. Krauss[2], Braxton D. Mitchell[3], Alan R. Shuldiner[3,4], and Alan D. Attie[1*]

[1]Department of Biochemistry, University of Wisconsin - Madison, Madison, WI, USA; [2]Department of Pediatrics, University of California - San Francisco, San Francisco, CA, USA; [3]Department of Medicine, University of Maryland School of Medicine, Baltimore, MD, USA; [4]Regeneron Genetics Center, Tarrytown, NY, USA.

**Table S1**. Linkage disequilibrium (LD) analysis for *SORT1* variants rs141749679 (K302E), rs149456022 (Q225H), and rs12740374.

| Variants | $r^2$ | D' |
|---|---|---|
| rs12740374 (non-coding) and rs141749679 (K302E) | <0.001 | 0.255 |
| rs12740374 (non-coding) and rs149456022 (Q225H) | 0.052 | 1.0 |
| rs141749679 (K302E) and rs149456022 (Q225H) | <0.001 | 0.019 |

**Table S2**. Association of *SORT1* variants with LDL-C in the Amish using a multivariate model where all three variants were analyzed together.

| Variant | Trait | Effect | *p*-value |
|---|---|---|---|
| rs141749679 (K302E) | LDL-C | 19.7 | 0.005 |
| rs149456022 (Q225H) | LDL-C | -4.2 | 0.07 |
| rs12740374 (non-coding) | LDL-C | -4.6 | $7.3 \times 10^{-9}$ |

**Table S3**. Association of *SORT1* variants with TC, LDL-C, non-HDL-C, HDL-C, and TG in trans-ancestry and ancestry-stratified phenome-wide association analysis (PheWAS) of aggregated GWAS results from the Global Lipids Genetics Consortium (GLGC) (30).

| **Ancestry** | ***rs141749679 (K302E)*** | | | ***rs149456022 (Q225H)*** | | | ***rs12740374 (non-coding)*** | | |
|---|---|---|---|---|---|---|---|---|---|
| **Trait** | **N** | **Beta** | ***p*** | **N** | **Beta** | ***p*** | **N** | **Beta** | ***p*** |
| *All* | | | | | | | | | |
| TC | 1,429,960 | 0.03 | 0.02 | 863,456 | -0.22 | 0.1 | 2,494,570 | -0.12 | $5\times10^{-324}$ |
| LDL-C | 1,346,170 | 0.04 | 0.002 | 841,766 | -0.10 | 0.02 | 2,641,730 | -0.15 | $5\times10^{-324}$ |
| non-HDL-C | 944,669 | 0.03 | 0.01 | 546,225 | -0.07 | 0.2 | 1,079,400 | -0.14 | $5\times10^{-324}$ |
| HDL-C | 1,351,590 | 0.003 | 0.6 | 807,865 | -0.03 | 0.5 | 3,018,430 | 0.03 | $7\times10^{-129}$ |
| TG | 1,167,780 | -0.02 | 0.04 | 848,051 | 0.04 | 0.4 | 2,988,740 | -0.01 | $3\times10^{-14}$ |
| *European* | | | | | | | | | |
| TC | 1,274,597 | 0.02 | 0.04 | 822,234 | -0.03 | 0.5 | 1,319,973 | -0.12 | $5\times10^{-324}$ |
| LDL-C | 1,193,087 | 0.04 | 0.003 | 798,434 | -0.08 | 0.1 | 1,231,254 | -0.15 | $5\times10^{-324}$ |
| non-HDL-C | 880,955 | 0.03 | 0.03 | 528,255 | -0.05 | 0.4 | 926,563 | -0.14 | $5\times10^{-324}$ |
| HDL-C | 1,199,551 | 0.0007 | 0.9 | 768,263 | -0.009 | 0.9 | 1,244,536 | 0.03 | $1\times10^{-97}$ |
| TG | 1,211,667 | -0.02 | 0.1 | 806,988 | 0.02 | 0.7 | 1,251,964 | -0.01 | $7\times10^{-12}$ |
| *Hispanic* | | | | | | | | | |
| TC | 41,222 | 0.07 | 0.5 | 41,222 | -0.10 | 0.1 | 35,478 | -0.16 | $1\times10^{-68}$ |
| LDL-C | 40,790 | 0.14 | 0.2 | 40,790 | -0.12 | 0.08 | 33,990 | -0.19 | $5\times10^{-87}$ |
| non-HDL-C | 17,256 | 0.09 | 0.6 | 17,970 | -0.12 | 0.2 | 10,433 | -0.19 | $1\times10^{-29}$ |
| HDL-C | 38,888 | 0.05 | 0.6 | 39,602 | -0.07 | 0.3 | 34,968 | 0.01 | 0.1 |
| TG | 42,503 | -0.05 | 0.6 | 41,063 | 0.06 | 0.4 | 37,273 | -0.001 | 0.9 |
| *African* | | | | | | | | | |
| TC | 79,154 | 0.14 | 0.3 | | | | 99,430 | -0.13 | $1\times10^{-145}$ |
| LDL-C | 79,088 | 0.08 | 0.5 | | | | 94,622 | -0.16 | $1\times10^{-187}$ |
| non-HDL-C | 16,488 | 0.50 | 0.02 | | | | 29,610 | -0.15 | $6\times10^{-54}$ |
| HDL-C | 78,211 | 0.24 | 0.05 | | | | 97,170 | 0.03 | $6\times10^{-8}$ |
| TG | 79,122 | -0.18 | 0.2 | | | | 96,341 | -0.01 | 0.01 |
| *South Asian* | | | | | | | | | |
| TC | 30,023 | -0.06 | 0.8 | | | | 40,962 | -0.14 | $2\times10^{-67}$ |
| LDL-C | 28,290 | -0.02 | 0.9 | | | | 40,472 | -0.17 | $6\times10^{-96}$ |
| non-HDL-C | 29,970 | -0.07 | 0.7 | | | | 38,314 | -0.16 | $2\times10^{-80}$ |
| HDL-C | 29,980 | -0.05 | 0.8 | | | | 40,172 | 0.05 | $3\times10^{-10}$ |
| TG | 29,571 | -0.06 | 0.5 | | | | 40,846 | -0.02 | 0.003 |

Beta reflects effect size for the transformed lipid level.

**Table S4**. Definitions and diameter ranges of lipoprotein particle classes and subclasses.

| Variable | Definition | Diameter range (Å) |
|---|---|---|
| *Major lipoprotein classes* | | |
| HDL | High density lipoproteins 3, 2a, and 2b | 76.50 - 145.00 |
| Midzone | Midzone between HDL and LDL | 145.01 - 180.00 |
| LDL | Low density lipoproteins IVc, IVb, IVa, IIIb, IIIa, IIb, IIa, I, and IDL2 | 180.01 - 250.00 |
| IDL | Intermediate density lipoprotein 1 | 250.01 - 296.00 |
| VLDL | Small, medium, and large very low density lipoproteins | 296.01 - 523.00 |
| *LDL subclasses* | | |
| vsLDL | Very small LDL (LDL IVc, IVb, IVa, and IIIb) | 180.01 - 208.20 |
| smLDL | Small LDL (LDL IIIa) | 208.21 - 214.10 |
| mdLDL | Medium LDL (LDL IIb) | 214.11 - 220.00 |
| lgLDL | Large LDL (LDL IIa, LDL I, and IDL2) | 220.01 - 250.00 |

Concentrations of lipoprotein particles were quantified by ion mobility, as previously described (20, 21). Particle numbers were pooled by summing the total number of particles within specific diameter ranges that approximately group the lipoproteins into separately defined classes or subclasses that have minimal methodologic and biologic overlap (22).

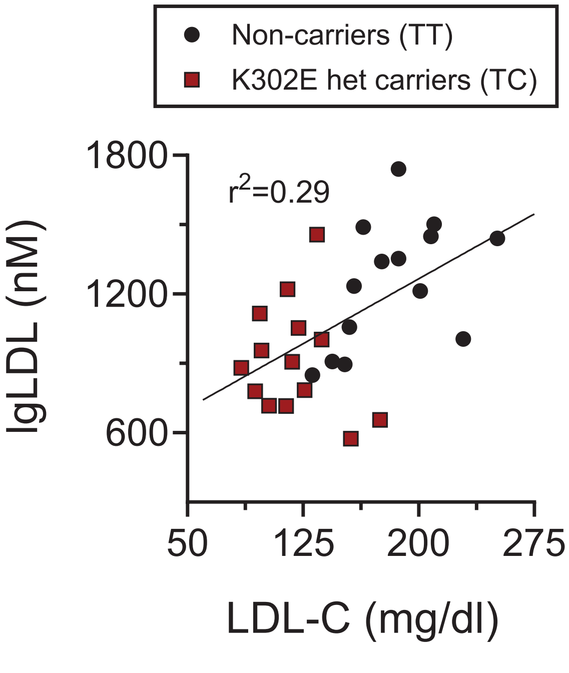


**Fig S1. LDL-C and lgLDL particle numbers are highly correlated in the Amish population**. Correlation between LDL-C and the concentration of large LDL (lgLDL) particles, as determined by ion mobility and defined in Table S4, in 14 Amish rs141749679 (K302E) heterozygote carriers and 14 age- and sex-matched non-carriers. Serum was collected after an overnight fast.
